# Versatile Construction of Single-Tailed Giant Surfactants with Hydrophobic Poly(ε-caprolactone) Tail and Hydrophilic POSS Head

**DOI:** 10.3390/polym11020311

**Published:** 2019-02-12

**Authors:** Qiangyu Qian, Jun Xu, Mingzu Zhang, Jinlin He, Peihong Ni

**Affiliations:** College of Chemistry, Chemical Engineering and Materials Science, State and Local Joint Engineering Laboratory for Novel Functional Polymeric Materials, Jiangsu Key Laboratory of Advanced Functional Polymer Design and Application, Suzhou Key Laboratory of Macromolecular Design and Precision Synthesis, Soochow University, Suzhou 215123, China; 20164209218@stu.suda.edu.cn (Q.Q.); xujunchemistry@163.com (J.X.); zhangmingzu@suda.edu.cn (M.Z.); phni@suda.edu.cn (P.N.)

**Keywords:** giant surfactant, thiol–ene “click” reaction, polyhedral oligomeric silsesquioxane (POSS), poly(ε-caprolactone), aqueous self-assembly

## Abstract

Giant surfactants refer to a new kind of amphiphile by incorporating functional molecular nanoparticles with polymer tails. As a size-amplified counterpart of small-molecule surfactants, they serve to bridge the gap between small-molecule surfactants and amphiphilic block copolymers. This work reports the design and synthesis of single-tailed giant surfactants carrying a hydrophobic poly(ε-caprolactone) (PCL) as the tail and a hydrophilic cage-like polyhedral oligomeric silsesquioxane (POSS) nanoparticle as the head. The modular synthetic strategy features an efficient “growing-from” and “click-modification” approach. Starting from a monohydroxyl and heptavinyl substituted POSS (VPOSS-OH), a PCL chain with controlled molecular weight and narrow polydispersity was first grown by the ring-opening polymerization (ROP) of ε-CL under the catalysis of stannous octoate, leading to a PCL chain end-capped with heptavinyl substituted POSS (VPOSS-PCL). To endow the POSS head with adjustable polarity and functionality, three kinds of hydrophilic groups, including hydroxyl groups, carboxylic acids, and amine groups, were installed to the periphery of POSS molecule by a high-efficiency thiol-ene “click” reaction. The compounds were fully characterized by NMR, gel permeation chromatography (GPC), MALDI-TOF mass spectrometry, and TGA analysis. In addition, the preliminary self-assembly study of these giant surfactants was also investigated by TEM and dynamic laser light scattering (DLS), which indicated that they can form spherical nanoparticles with different diameters in aqueous solution. This work affords a straightforward and versatile way for synthesizing single-tailed giant surfactants with diverse head surface functionalities.

## 1. Introduction

Amphiphiles refer to a kind of molecules containing chemically distinct segments, such as hydrophobic and hydrophilic parts, linked with chemical or supramolecular bonds. Traditionally, amphiphiles were generally accepted as small-molecule surfactants composed of a hydrophilic polar head and a hydrophobic alkyl chain, which have been broadly used in our daily life, including detergents, dispersants, cosmetics, and pharmaceutical excipients [1]. Afterwards, the region of amphiphiles was extended to amphiphilic block copolymers consisting of hydrophilic and hydrophobic polymeric chains. They have drawn tremendous attention in the past three decades because of their promising applications in various fields [2,3,4,5,6]. It is expected that an integration of both characteristics of small-molecule surfactants and amphiphilic block copolymers would expand the scope of amphiphiles and produce new properties.

In recent years, an emerging category of amphiphiles, named giant surfactants, has been constructed and widely studied through covalently connecting functionalized molecular nanoparticles with polymeric tails [7,8,9,10,11,12,13,14]. They have obtained much attention due to their multivalent nature, promising physical/chemical features, and unique self-assembling behaviors, both in bulk and solution states [10,11,15,16]. As a size-amplified analogue of small-molecule surfactants, giant surfactants serve to fill the niche between small-molecule surfactants and amphiphilic block copolymers. Just like the small-molecule surfactants, the giant surfactants can also be fabricated into various topologies, including single-head/single-tail, single-head/two-tails, or more complex two-head/two-tail (gemini-like), two heads in each terminal of one tail (bolaform-like), and even multiheaded/multitailed style [10]. On the other hand, giant surfactants obviously provide much more space for engineering chemical structures than their small-molecule counterparts. For instance, the polymeric tails can be of different topologies (e.g., linear, cyclic, or branched) or of distinct compositions, while the molecular nanoparticle heads can be modified with various functionalities, such as ionic, non-ionic, bioactive, etc. Therefore, all of these characteristics endow giant surfactants with sophisticated structures and thus result in more complicated self-assembly behaviors, as well as adjustable functional properties [8].

Specifically, the molecular nanoparticles are recognized as unique nanoscale building blocks with fixed shape and volume, precisely-defined chemical structures, as well as versatile surface functionalities, which notably distinguish giant surfactants from the other two typical amphiphiles [9]. To date, representative molecular nanoparticles that have been developed include folded globular proteins [12,17] and cage-like compounds, such as polyhedral oligomeric silsesquioxanes (POSS) [18,19], fullerenes [20], as well as polyoxometalates (POM) [21]. Thereinto, POSS-based molecular nanoparticles have been intensively employed for making giant surfactants, possibly because of the following considerations: (1) POSS molecule is probably the smallest shape-persistent cage-like silsesquioxane nanoparticles with a diameter of about 1.0 nm; (2) the rigid three-dimensional structure of the POSS molecule is relatively stable in common conditions; and (3) it is easy to modify both the cage structures (e.g., T_8_ and T_12_) and the surface functionalities (e.g., mono-, multi-, regio-, homo-, or hetero-functionalized) of POSS molecules. With the aid of living/controlled polymerizations and the powerful “click” reactions [22,23], a variety of POSS-based giant surfactants bearing different kinds of architecture have been constructed [24,25,26,27,28,29,30,31,32]. It has been shown that, by using such combined methodologies, several crucial molecular parameters of giant surfactants could be manipulated, such as overall molecular weights, surface functionalities, polydispersity, and weight fraction of tails/heads. Recently, we have constructed a library of double-chain giant surfactant region-isomers composed of a hydrophilic hydroxyl-functionalized POSS head and two hydrophobic polystyrene tails with various molecular weights tethered in ortho-, meta-, and para-configurations [33,34]. It was surprising to find that such a minute difference in regio-configuration influences a lot on the self-assembly behaviors of these giant surfactants, which identify regio-chemistry as an additional important factor in tuning the self-assembly of giant surfactants.

**Scheme 1:**
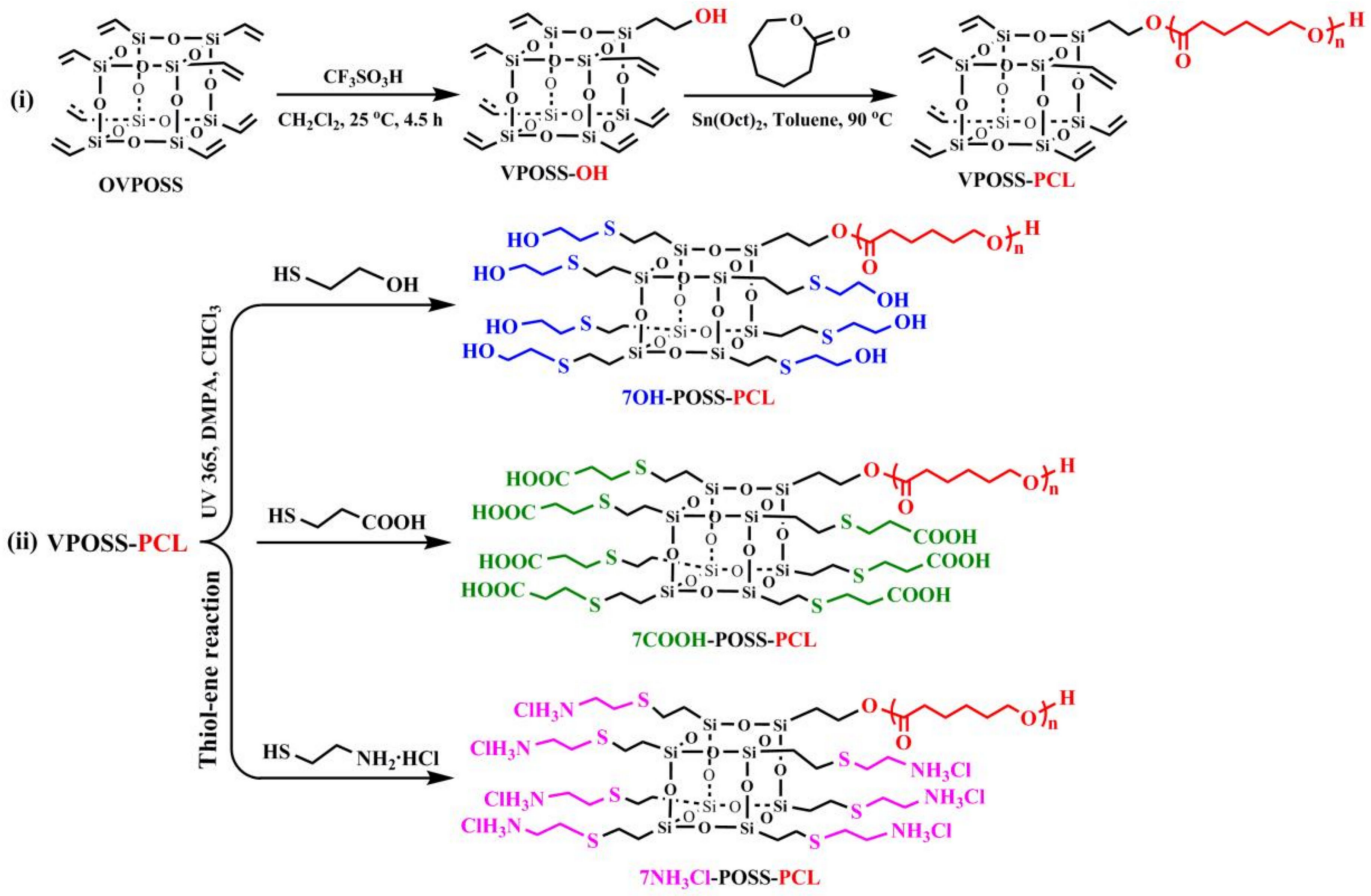
Synthetic routes of single-tailed giant surfactants 7R-polyhedral oligomeric silsesquioxane (POSS)-poly(ε-caprolactone) (PCL) (R = OH, COOH, and NH_3_Cl) via a combination of ring-opening polymerization (ROP) and thiol-ene “click” reaction.

Herein, we report the model preparation of single-tailed giant surfactants on the basis of poly(ε-caprolactone) (PCL) end-capped with functional POSS (7R-POSS-PCL, R = OH, COOH, and NH_3_Cl) using a sequential strategy of polymeric chain growth and POSS head modification. As shown in Scheme 1, PCL carrying seven vinyl groups (VPOSS-PCL) was first grown from VPOSS-OH through stannous octoate-catalyzed ring-opening polymerization (ROP) with controlled molecular weights and narrow polydispersities [35]. As a representative aliphatic polyester, PCL has been widely studied and applied in various biomedical fields due to its excellent biocompatibility, superior synthetic versatility, and flexible mechanical properties. Subsequently, the POSS headgroup was modified with diverse functionalities, including hydroxyl groups, carboxylic acids, and amine groups, via thiol-ene “click” reaction in high efficiency. As anticipated, by means of the controlled ROP reaction and powerful thiol-ene “click” chemistry, this universal method should be readily available to other polymerizable monomers and periphery functionalities.

## 2. Materials and Methods

### 2.1. Materials

Toluene (A.R., Sinopharm Chemical Reagent, Shanghai, China) and ε-caprolactone (ε-CL, 99%, Acros, Geel, Belgium) were dried by stirring in CaH_2_ powder for 24 h at room temperature and distilled under reduced pressure before use. Dichloromethane (CH_2_Cl_2_, A.R., Sinopharm Chemical Reagent, Shanghai, China) was refluxed with CaH_2_ powder and distilled before use. OctavinylPOSS (OVPOSS, 97%, Shanghai Gileader Advanced Material Technology, Shanghai, China) was eluted with CH_2_Cl_2_ (A.R., Sinopharm Chemical Reagent, Shanghai, China) to remove polar impurities, condensed by rotation distillation, and, finally dried in a vacuum oven to give a white powder. Stannous octoate (Sn(Oct)_2_, 95%, Sigma-Aldrich, Saint Louis, MI, USA) was first fractionally distilled under reduced pressure and then diluted with anhydrous toluene to make a solution with a concentration of 0.5 g/mL in a glove box. Triflic acid (99%, J&K Scientific, Shanghai, China), 2,2-dimethoxy-2-phenylacetophenone (DMPA, 98%, TCI, Shanghai, China), 2-mercaptoethanol (98%, Acros, Geel, Belgium), 3-mercaptopropionic acid (99%, Acros, Geel, Belgium), and cysteamine hydrochloride (98%, Acros, Geel, Belgium) were used as received. Milli-Q ultrapure water (18.2 MΩ cm at 25 °C) was generated by a water purification system (Simplicity UV, Millipore, Shanghai, China). All the other chemicals (Sinopharm Chemical Reagent, Shanghai, China) were analytical reagents and used as received, unless otherwise mentioned.

### 2.2. Synthesis of Monohydroxyl Heptavinyl Substituted POSS (VPOSS-OH)

The monohydroxyl-functionalized heptavinyl POSS (VPOSS-OH) was prepared from commercially available OVPOSS, according to the literature method developed by Feher et al., after some modification [36]. The detailed synthetic procedure is listed as follows: Before reactions, the glassware, including stirring bars, were dried in an oven at 120 °C. OVPOSS (15 g, 23.7 mmol) was added to a round-bottom flask containing 200 mL of dry CH_2_Cl_2_ and stirred for complete dissolution. After adding triflic acid (4.2 mL, 23.7 mmol) to the above solution, the reaction was conducted at 25 °C for 4.5 h. After that, the mixture was washed three times with saturated aqueous NaHCO_3_, and the collected organic phase was then mixed with 30 mL of acetone/water (v/v = 4:1) and the hydrolysis was performed at 25 °C for another 12 h. After the reaction, the organic phase was collected and dried with anhydrous Na_2_SO_4_, and the crude product was obtained after evaporation of the solvent. Column chromatography on silica with CH_2_Cl_2_/petroleum ether as the eluent afforded VPOSS-OH as a white solid (2.1 g, yield: 13%).

### 2.3. Preparation of Poly(ε-caprolactone) End-Capped with Heptavinyl Substituted POSS (VPOSS-PCL)

The poly(ε-caprolactone) end-capped with the heptavinyl substituted POSS head (VPOSS-PCL) was obtained by ROP of the ε-CL using VPOSS-OH as the initiator and Sn(Oct)_2_ as the catalyst [35]. Briefly, VPOSS-OH (0.20 g, 0.31 mmol) was added to a 50 mL of Schlenk flask, which was then heated at 50 °C under a high vacuum to remove the possible residual moisture. After that, 15 mL of dry toluene was transferred to the reactor to dissolve the initiator under stirring. To this solution, ε-CL (1.71 g, 15 mmol) and Sn(Oct)_2_ (0.3 mL, 0.15 mmol, 0.5 g/mL solution in dry toluene) were added by syringe under a dry nitrogen atmosphere. After degassing the solution by three exhausting–refilling nitrogen cycles, the mixture was kept stirring at 90 °C for 8 h. Afterwards, the viscous solution was concentrated and precipitated in cold methanol thrice. The precipitate was collected and dried at 25 °C under a vacuum to a constant weight, resulting in the product of VPOSS-PCL as a white powder (1.64 g, yield: 86%).

### 2.4. Typical Procedure for Synthesizing Single-Tailed Giant Surfactants (7R-POSS-PCL)

The single-tailed giant surfactants (7R-POSS-PCL, R = OH, COOH, and NH_3_Cl) were prepared by the respective reaction between VPOSS-PCL and functional thiols (2-mercaptoethanol, 3-mercaptopropionic acid, and cysteamine hydrochloride) using a UV-irradiated thiol-ene chemistry [37,38]. The representative procedure is described as follows: In a round-bottom quartz flask, VPOSS-PCL (0.15 g, 0.03 mmol), 2-mercaptoethanol (98.3 mg, 1.26 mmol), and DMPA (5.4 mg, 0.021 mmol) were dissolved in 2 mL of CHCl_3_. After irradiation with UV 365 nm at room temperature for 20 min, the mixture was purified by repeated precipitation in cold methanol. The white solid was collected by centrifugation and dried under a high vacuum for 24 h to give the 7OH-POSS-PCL (0.14 g, yield: 84%). The other two single-tailed giant surfactants, i.e., 7COOH-POSS-PCL and 7NH_3_Cl-POSS-PCL, were prepared using a similar protocol for synthesizing 7OH-POSS-PCL.

### 2.5. Self-Assembly of Giant Surfactants in Aqueous Solution

The nanoparticles self-assembled by single-tailed giant surfactants 7R-POSS-PCL in aqueous solution were prepared by a dialysis method. Briefly, 5 mg of polymer sample was dissolved in 1.5 mL dimethylformamide (DMF) in a round-bottom flask, and it was stirred for several hours to achieve complete dissolution. Subsequently, 15 mL of Milli-Q water was added dropwise during a period of 2 h, using an auto-sampling system under moderate stirring. After that, the nanoparticle solution was dialyzed (MWCO 3500) against Milli-Q water for 24 h to remove DMF. The dialysis medium was changed six times during the process. Lastly, the solution was diluted to 25 mL with Milli-Q water to a desired concentration. Dust particles were removed by filtering each solution through an Φ 450 nm microfilter before measurements. The average particle sizes and size distributions of nanoparticles were determined by a Malvern dynamic laser light scattering (DLS, Zetasizer Nano-ZS, Malvern, UK) instrument. The morphologies of the nanoparticles were observed using TEM (HT7700, Hitachi, Tokyo, Japan), operated at an accelerating voltage of 120 kV. The carbon-coated copper grid was placed on the bottom of a glass cell, which was then immediately inserted into liquid nitrogen. After that, 10 μL of the solution was dripped on the grid and the frozen solvent was directly removed in a freeze dryer. The morphologies were then imaged on a normal TEM instrument at room temperature.

### 2.6. Characterizations

^1^H NMR and ^13^C NMR analyses were conducted on a 400 MHz NMR instrument (INOVA-400, Varian, Palo Alto, CA, USA) with CDCl_3_ or *d*_6_-DMSO as the solvents and tetramethylsilane (TMS) as the internal reference. The number-average molecular weights (*M*_n, GPC_) and molecular weight distributions (*M*_w_/*M*_n_) of polymers were recorded on a gel permeation chromatography (GPC) instrument (HLC-8320, TOSOH, Tokyo, Japan), which was equipped with a refractive index and UV detectors using two TSKgel SuperMultiporeHZ-N (4.6 × 150 mm, 3.0 μm beads size) columns arranged in a series. It can separate polymers in the molecular weight range of 500–1.9 × 10^5^ g/mol. TGA of polymers was performed on a Discovery instrument (TA, New Castle, DE, USA) under a nitrogen atmosphere, and the data were recorded over a temperature range of 30–800 °C at a heating rate of 10 °C/min. MALDI-TOF mass spectra were measured on an UltrafleXtreme MALDI-TOF mass spectrometer (Bruker, Kalsruhe, Germany) equipped with a 1 kHz smart beam-II laser. Before each measurement, the instrument was calibrated by external poly(methyl methacrylate) (PMMA) or polystyrene (PS) standards with desired molecular weights. Trans-2-[3-(4-tert-butylphenyl)-2-methyl-2-propenylidene]-malononitrile (DCTB, >99%, Sigma-Aldrich, Saint Louis, MI, USA) was used as the matrix and prepared in CHCl_3_ with a concentration of 20 mg/mL. Sodium trifluoroacetate (CF_3_COONa, >99%, Sigma-Aldrich, Saint Louis, MI, USA) served as the cationizing agent and was dissolved in anhydrous ethanol to make a solution with a concentration of 10 mg/mL. The solutions of matrix and CF_3_COONa were mixed in a ratio of 10/1 (v/v). The polymers were dissolved in CHCl_3_ with a concentration of 10 mg/mL. The sample preparation included depositing 0.5 μL of mixture solution of matrix/salt on the wells of a 384-well ground-steel plate, allowing the spots to dry completely, and then adding 0.5 μL of each sample solution on a spot of dry matrix/salt before adding another 0.5 μL of matrix/salt mixture solution on the top of the dry sample. After the solvent was completely evaporated, the plate was inserted in the MALDI mass spectrometer. The attenuation of Nd:YAG laser was adjusted to minimize undesirable polymer fragmentation and to maximize the sensitivity. Data analyses were conducted with Bruker’s flexAnalysis software (Bruker Daltonics, Bremen, Germany).

## 3. Results and Discussion

### 3.1. Structure Characterization of Functional Initiator VPOSS-OH

**Figure 1:**
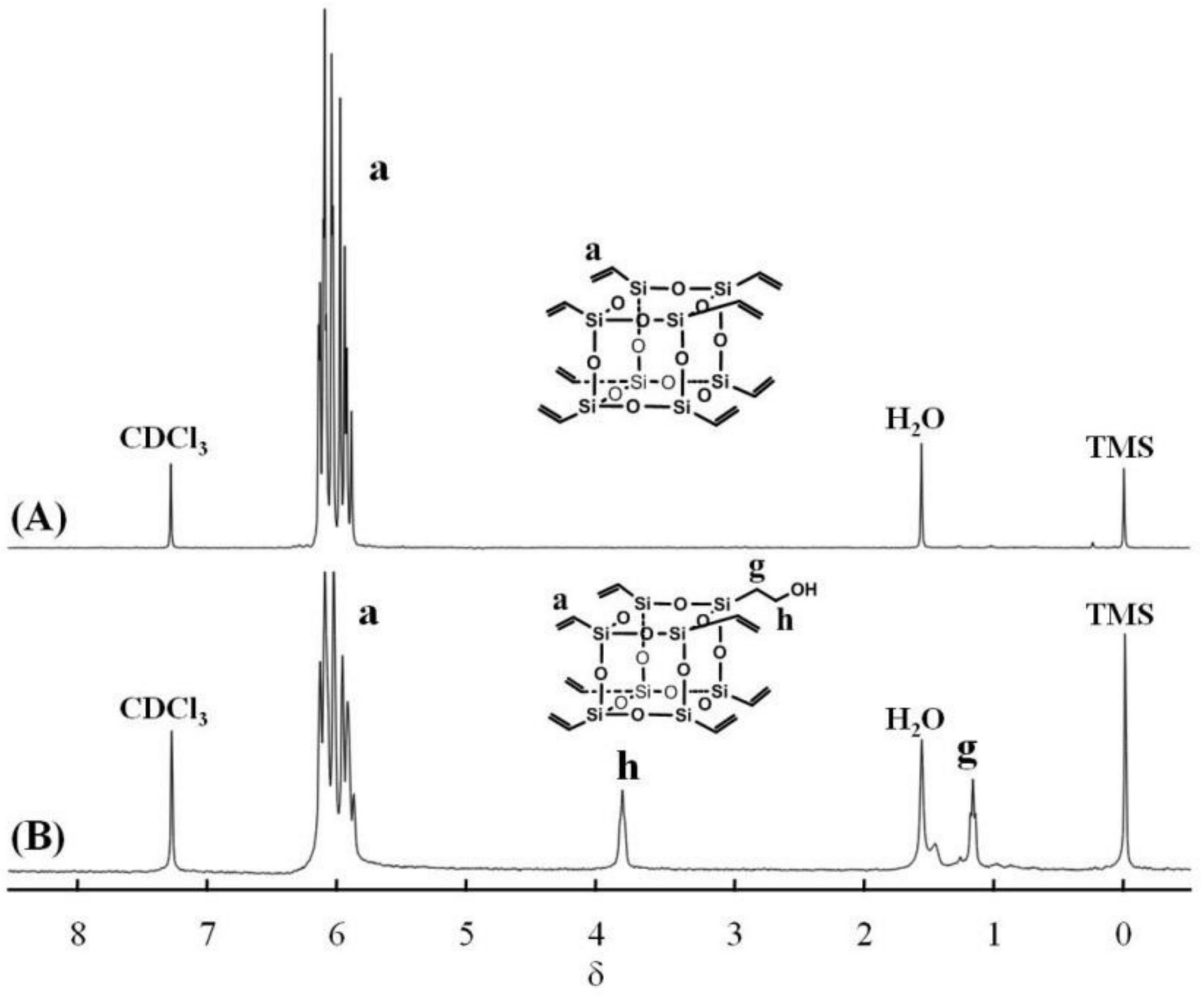
^1^H NMR spectra of (**A**) OctavinylPOSS (OVPOSS) and (**B**) VPOSS-OH in CDCl_3_.

**Figure 2:**
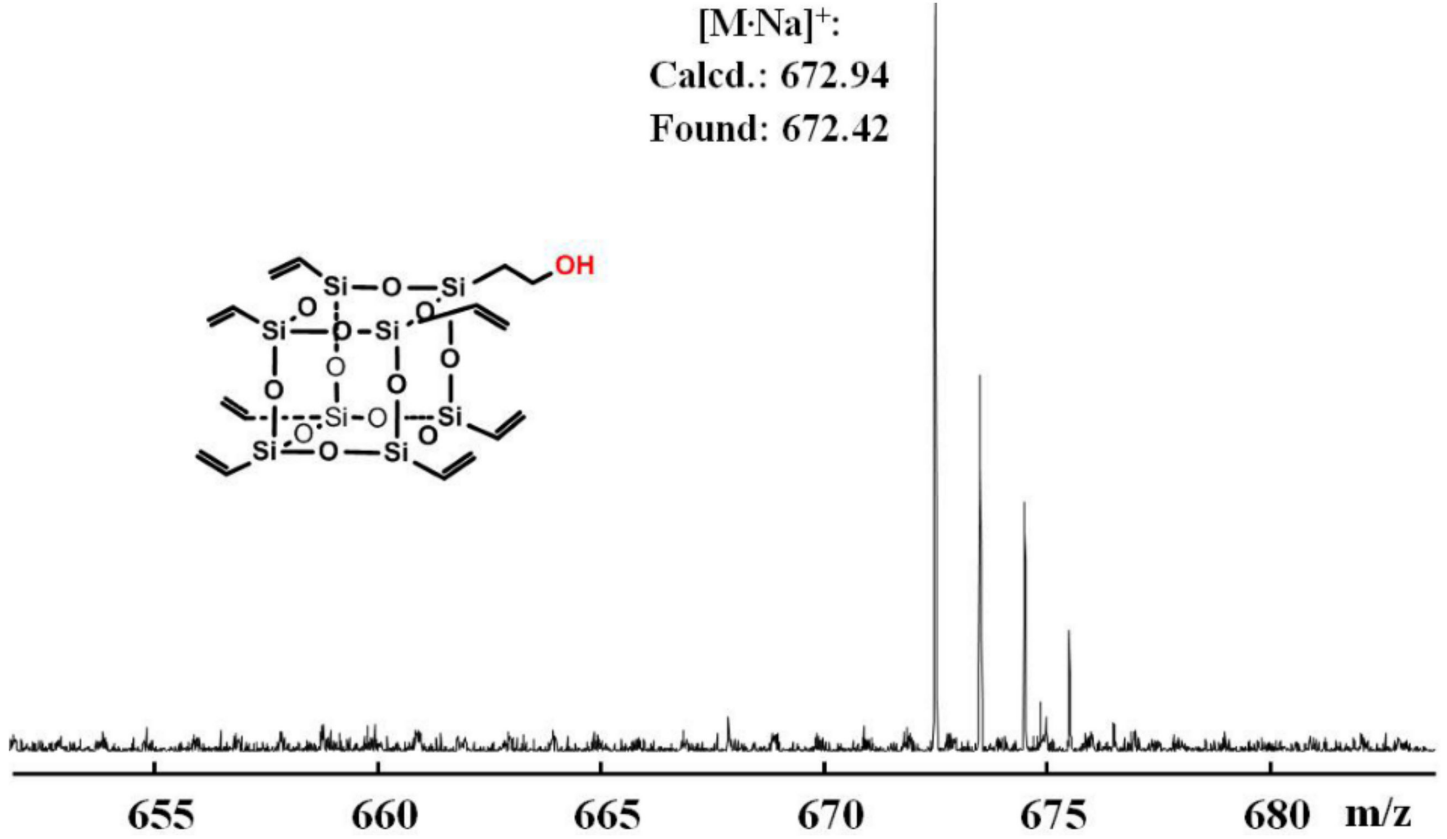
MALDI-TOF mass spectrometry (MS) spectrum of monohydroxyl heptavinyl substituted POSS (VPOSS-OH).

The whole synthetic approach shown in Scheme 1 was designed in an effort to achieve giant surfactants with desirable structure and diverse functions using simple reactions and readily available starting chemicals. From the commercially available octavinylPOSS, the monohydroxyl and heptavinyl functionalized VPOSS-OH was easily obtained in an acceptable yield, with all the characterizations consistent with the literature. As shown in Figure 1, the characteristic signals of vinyl protons appeared at δ 5.75–6.25 ppm. After the formation of VPOSS-OH, most of the vinyl protons remained at the same chemical shift and two new peaks could be found at δ 1.1 ppm and δ 3.8 ppm that were ascribed to the methylenes adjacent to hydroxyl group. Moreover, the molecule was also characterized by MALDI-TOF mass spectroscopy, and the result is displayed in Figure 2. It was found that the observed molecular weight (*m/z* = 672.42 Da) for (M∙Na)^+^ (C_16_H_26_O_13_Si_8_Na^+^) was in excellent agreement with the calculated one (*m/z* = 672.94 Da). 

### 3.2. Synthesis and Characterization of VPOSS-PCL

The versatile hydroxyl group in VPOSS-OH was able to initiate ROP of various cyclic monomers under various conditions. It could also be further transformed to other functional initiating groups, such as tosylate for living cationic polymerization, halide group for atom transfer radical polymerization (ATRP) reaction, or chain transfer agents for reversible addition–fragmentation chain transfer (RAFT) polymerization. Herein, ROP reaction of ε-caprolactone was selected as the model system and this method should also be applicable to other functional cyclic monomers, such as lactides, carbonates, and phosphoesters. The ROP reaction of ε-CL under the catalysis of Sn(Oct)_2_ has been well studied to prepare PCL with controlled molecular weight and narrow polydispersity. The polymerization was performed at 90 °C in toluene for 8 h using 0.5 equiv. of Sn(Oct)_2_ to VPOSS-OH, and the polymer was purified by repeated precipitation in methanol to remove the catalyst and unreacted monomer. VPOSS-PCL is obtained as a white powder with a yield of around 86% and readily soluble in most organic solvents. The molecular weights of VPOSS-PCL can be easily tuned by varying the feeding ratio of ε-CL to VPOSS-OH. The polymers were fully characterized by various techniques to confirm the structure and purity.

**Figure 3:**
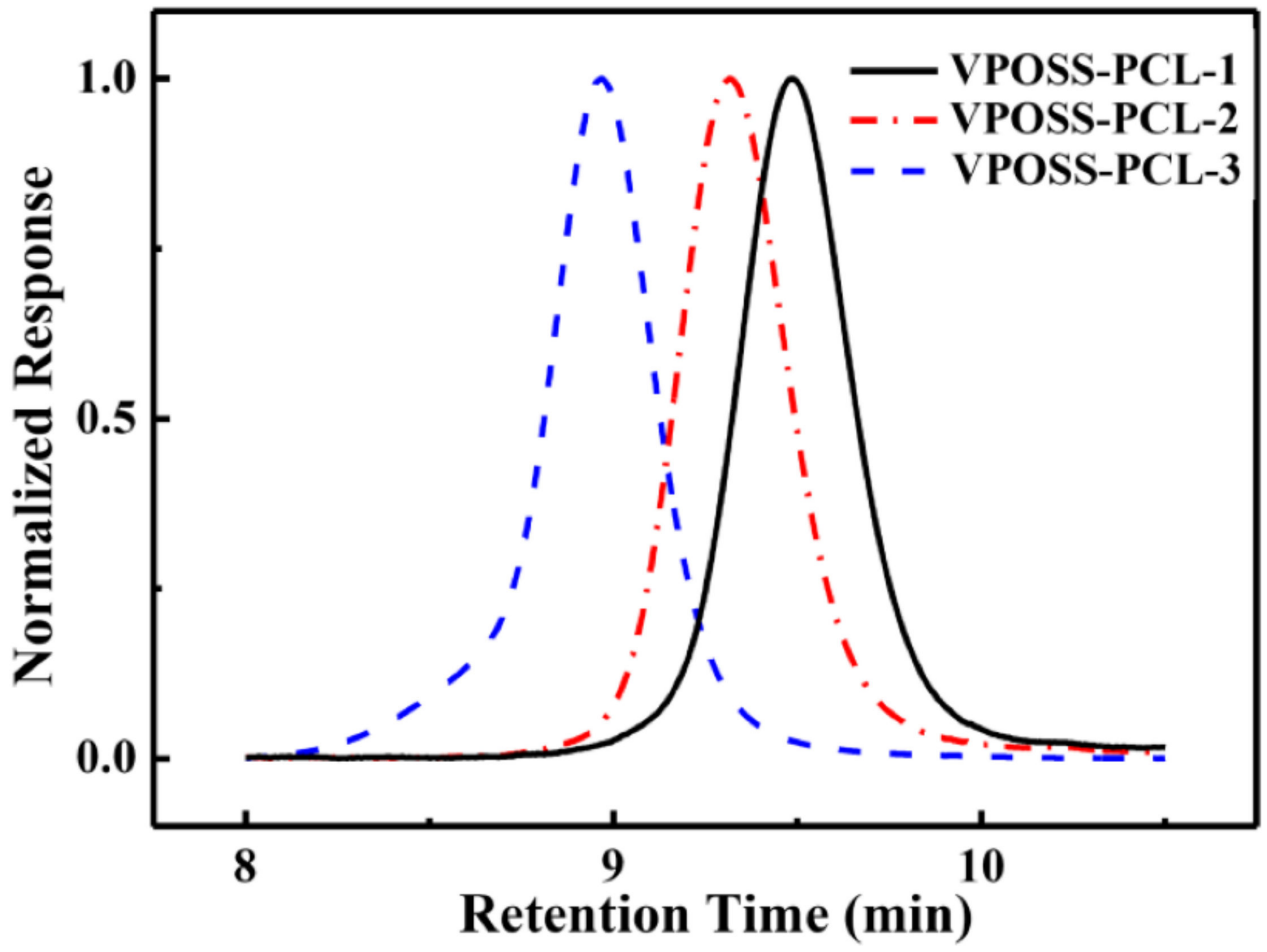
**Gel permeation chromatography** (GPC) traces of VPOSS-PCL samples (VPOSS-PCL-1: *M*_n, GPC_ = 3800 g/mol, *M*_w_/*M*_n_ = 1.09; VPOSS-PCL-2: *M*_n, GPC_ = 5000 g/mol, *M*_w_/*M*_n_ = 1.07; VPOSS-PCL-3: *M*_n, GPC_ = 7700 g/mol, *M*_w_/*M*_n_ = 1.11).

**Figure 4:**
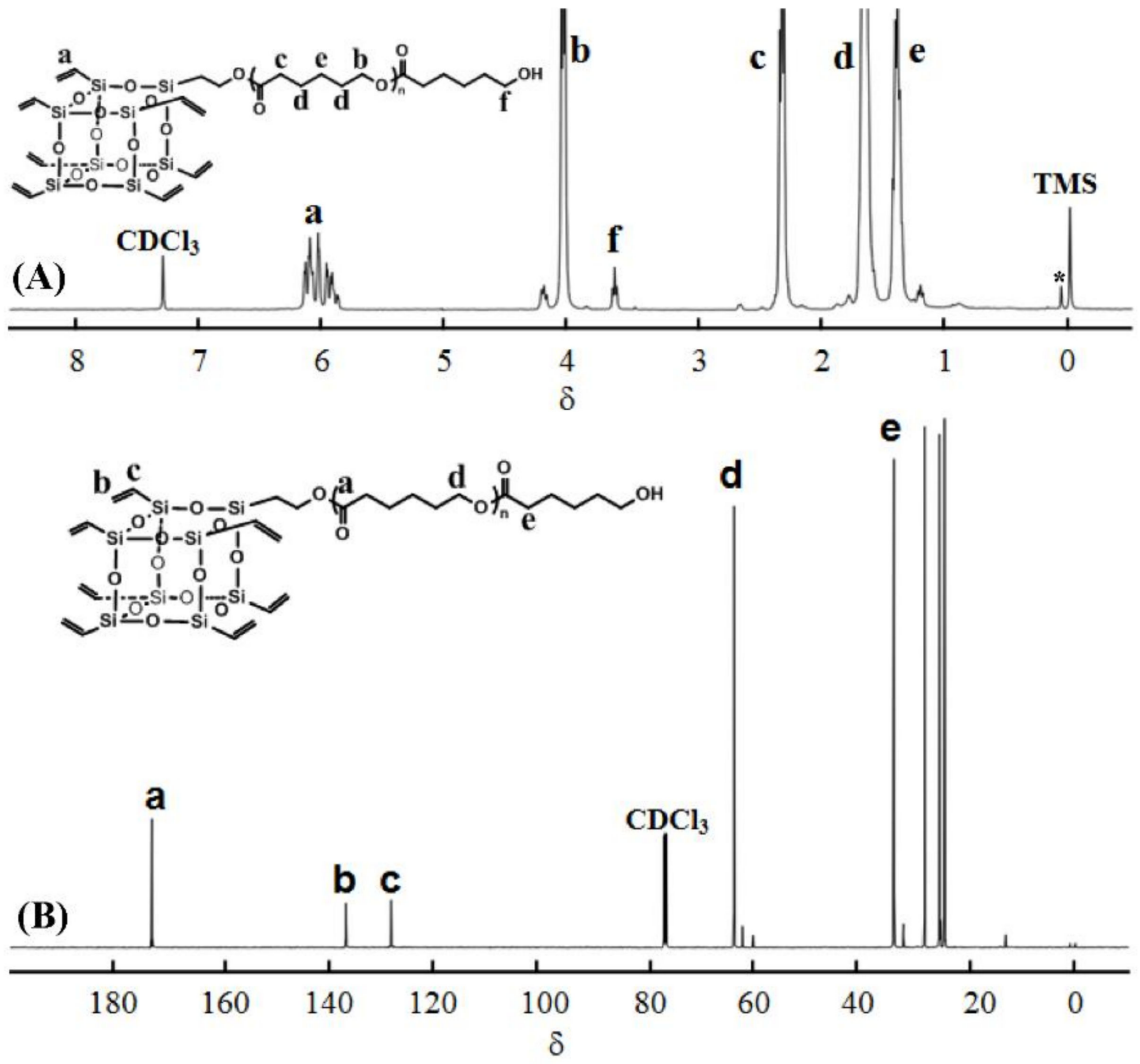
^1^H NMR spectrum (**A**) and ^13^C NMR spectrum (**B**) of VPOSS-PCL-3 in CDCl_3_. Asterisk represents resonance from residual silicone grease.

**Figure 5:**
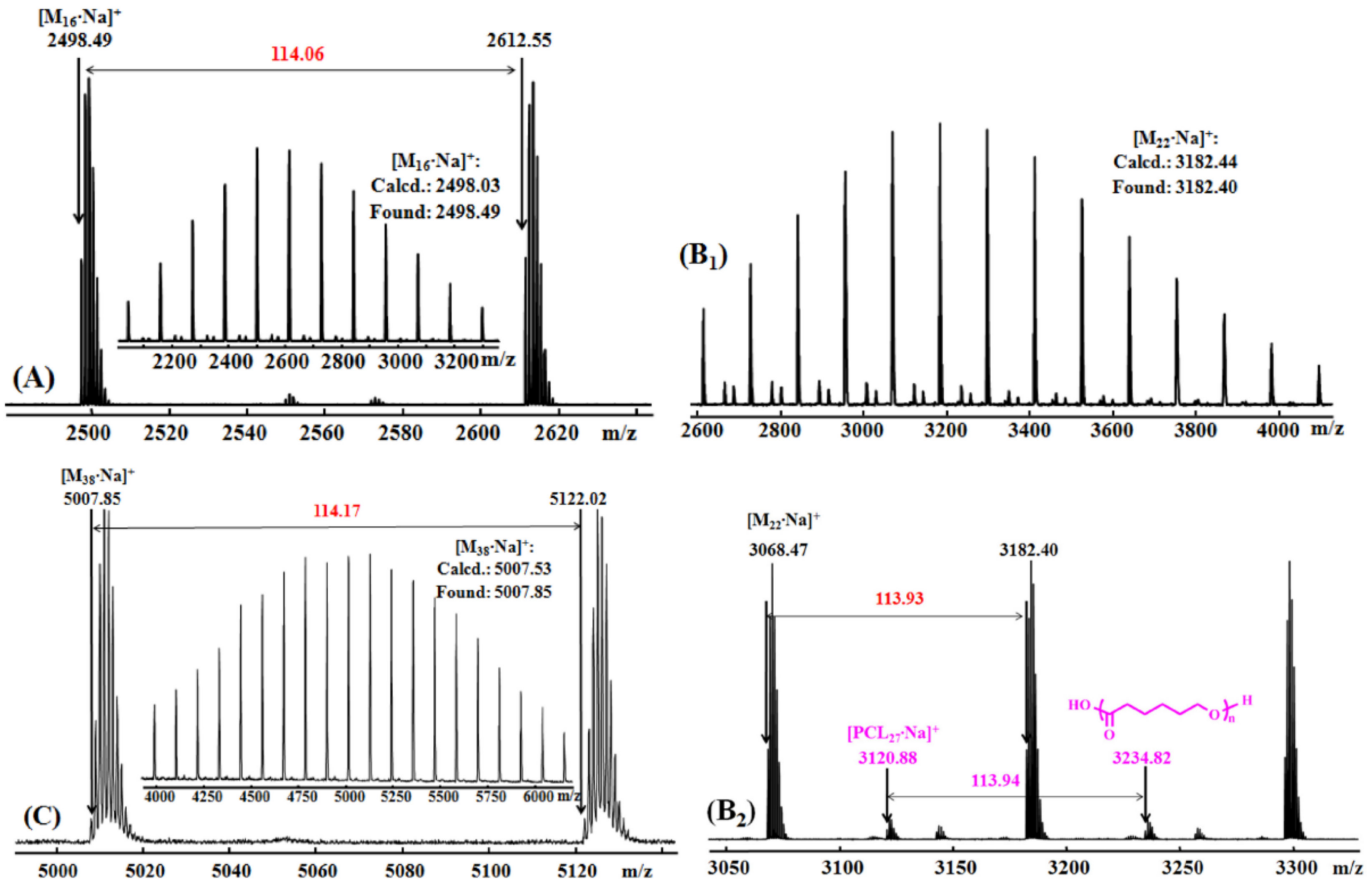
MALDI-TOF MS spectra of (**A**) VPOSS-PCL-1, (**B**) VPOSS-PCL-2 (B_2_ is the enlarged image of B_1_), and (**C**) VPOSS-PCL-3.

In the GPC curves shown in Figure 3, three VPOSS-PCL samples with different molecular weights and relatively narrow polydispersities (*M*_w_/*M*_n_ around 1.1) were obtained. All the GPC curves showed a unimodal and symmetrical pattern, and the high-molecular-weight samples (VPOSS-PCL-2 and VPOSS-PCL-3) displayed a major distribution that shifts towards the higher molecular weight side. In the typical ^1^H NMR spectrum (Figure 4A) of VPOSS-PCL-3, the vinyl protons remain at δ 5.75–6.25 ppm (peak a), while the characteristic protons ascribed to methylenes in the PCL backbone showed at peaks b–f, confirming the successful linking of VPOSS with the PCL chain. This was also affirmed by the observation of carbons from both the VPOSS and PCL chain in the ^13^C NMR spectrum (Figure 4B). Moreover, the integration ratio of peaks a and c in Figure 4A was used to calculate the molecular weight of VPOSS-PCL, and the results are listed in Table 1. The well-defined structure of VPOSS-PCL was also confirmed by the MALDI-TOF mass spectra. It is clear from Figure 5 that all three VPOSS-PCL samples displayed one single molecular weight distribution, and the observed molecular weight was in excellent agreement with the calculated one. For example, for VPOSS-PCL-1, the observed *m*/*z* value (2498.49 Da) for (M_16_·Na)^+^ with the formula of C_112_H_186_O_45_Si_8_Na^+^ agreed well with the calculated one (*m*/*z* 2498.03 Da). The same agreement was also found for the other two VPOSS-PCL samples. In addition, the mass difference between all adjacent two peaks was very close to the caprolactone repeating unit (*m*/*z* 114.07 Da). On the other hand, it needs to be pointed out that a minor distribution could be found in VPOSS-PCL-2, shown in Figure 5B_1_. From the enlarged view shown in Figure 5B_2_, one could find the minor distribution was probably ascribed to the PCL initiated by residual water during polymerization. Nevertheless, the chemical structure of VPOSS-PCL was thus unambiguously confirmed, and the sample was ready for further modification. Particularly, no fractionation was required in this polymerization process, and this facilitated easy synthesis of gram quantities of polymer samples.

### 3.3. Synthesis and Characterization of Single-Tailed Giant Surfactants 7R-POSS-PCL

**Figure 6:**
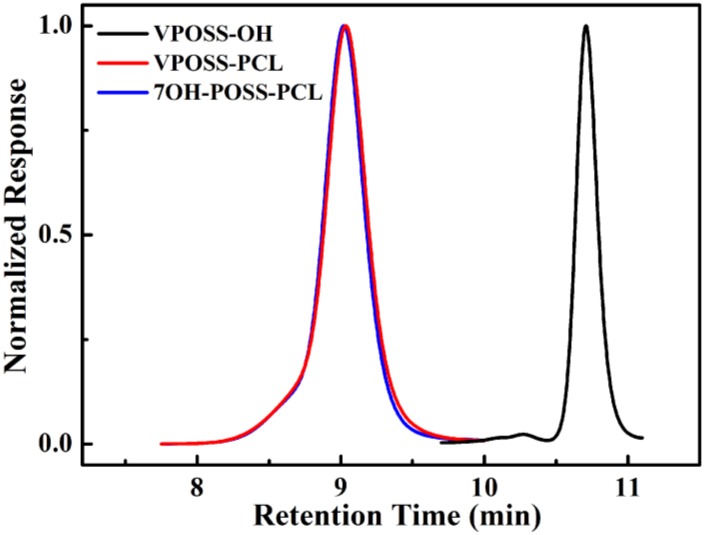
GPC traces of VPOSS-OH (*M*_n, GPC_ = 900 g/mol, *M*_w_/*M*_n_ = 1.14), VPOSS-PCL-3 (*M*_n, GPC_ = 7700 g/mol, *M*_w_/*M*_n_ = 1.11), and 7OH-POSS-PCL-3 (*M*_n, GPC_ = 7900 g/mol, *M*_w_/*M*_n_ = 1.10).

As a well-established methodology, thiol-ene “click” reaction has been broadly applied for various functionalizations [37]. In particular, it is quite powerful for situations when multiple modifications or sites of poor reactivity are involved in polymers. In this case, in order to tune the properties of the POSS headgroups, a variety of functional groups (-OH, -COOH, -NH_3_Cl) were successfully introduced to the POSS head. The synthesis was rapid and straightforward from commercially available starting materials, and it was sure that this model functionalization could also be extended to other systems as needed. The functional polymers were characterized by various techniques. In representative GPC curves (Figure 6), the elution profile of 7OH-POSS-PCL-3 was basically the same as that of VPOSS-PCL-3, indicating that the modification with very small functional groups on such a rigid POSS head did not affect the overall hydrodynamic volume a lot. Unfortunately, the other two sets of samples could not be used for GPC measurements since the polar ionic groups have very strong interaction with the separation column. 

**Figure 7:**
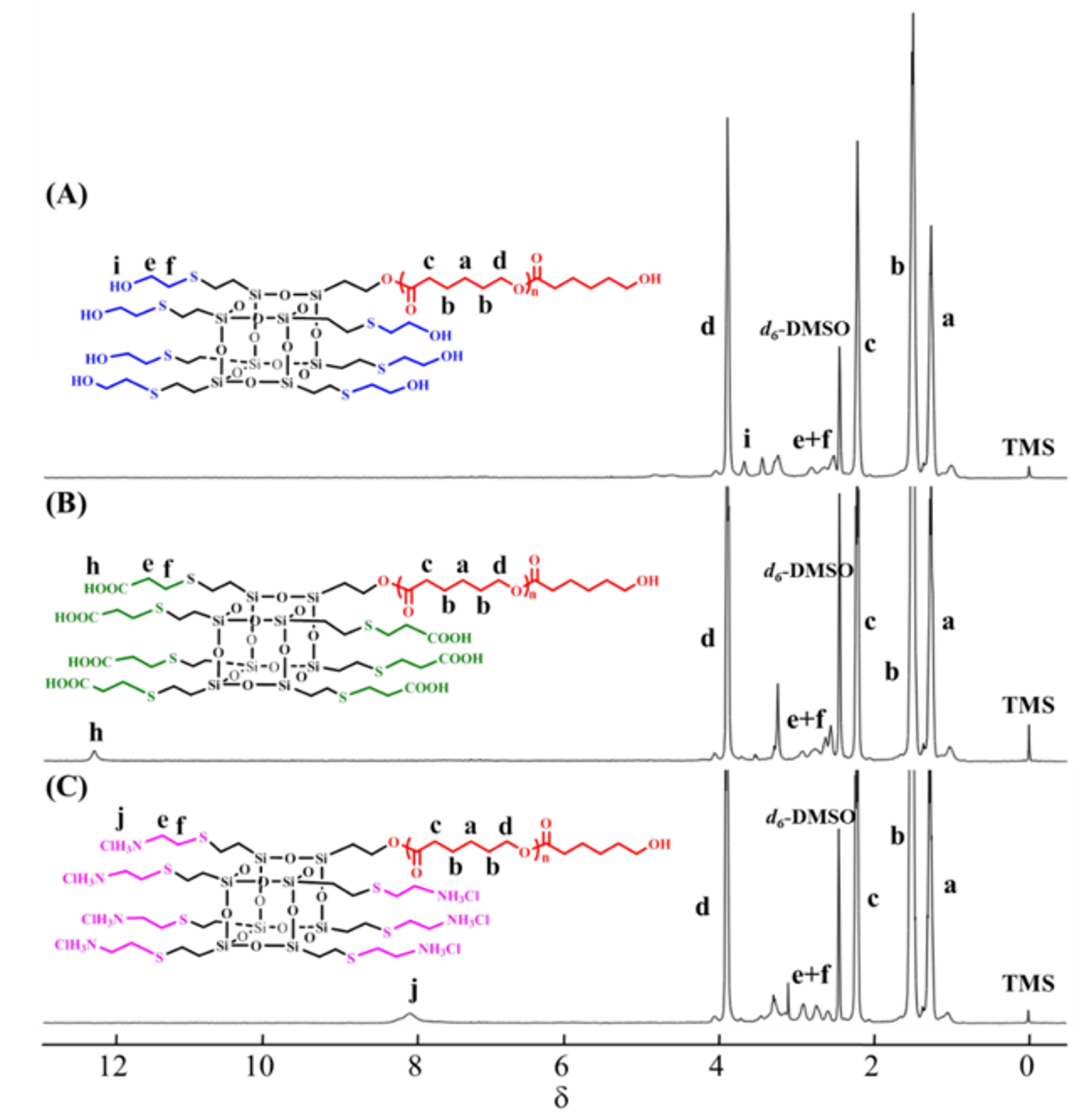
^1^H NMR spectra of (**A**) 7OH-POSS-PCL-3, (**B**) 7COOH-POSS-PCL-3, and (**C**) 7NH_3_Cl-POSS-PCL-3 in *d*_6_-DMSO.

**Figure 8:**
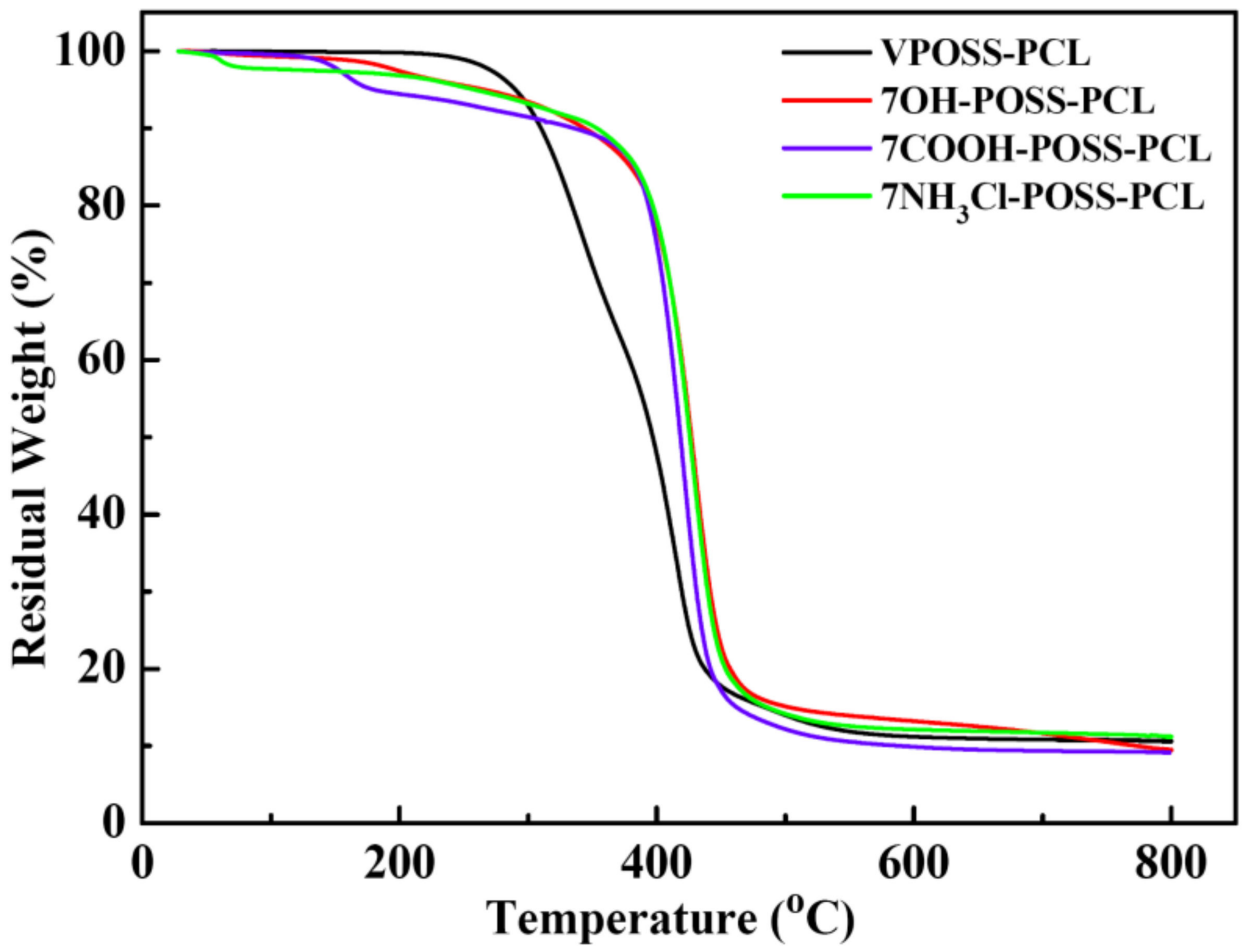
TGA thermograms of VPOSS-PCL-3, as well as the corresponding giant surfactants 7OH-POSS-PCL-3, 7COOH-POSS-PCL-3, and 7NH_3_Cl-POSS-PCL-3.

By means of the ^1^H NMR analysis (Figure 7), the successful ligation of the VPOSS headgroup by different small molecules was proven by the complete disappearance of vinyl proton resonances at δ 5.75–6.25 ppm (peak a in Figure 4A) and the new appearance of thio-ether methylene linkages at δ 2.5–3.7 ppm in the ^1^H NMR spectra (Figure 7). The chemical shifts agreed well with that of the reported analogues. In particular, the proton resonances of –OH, –COOH, and –NH_3_Cl were shown at δ 3.8 ppm, 12.1 ppm, and 8.1 ppm, respectively. Moreover, TGA test was performed to further characterize the polymers, and the results are shown in Figure 8. It was clearly observed that the starting degradation temperature of VPOSS-PCL-3 was around 250 °C, while the values for 7OH-POSS-PCL-3, 7COOH-POSS-PCL-3, and 7NH_3_Cl-POSS-PCL-3 were decreased to lower than 200 °C due to the incorporation of polar small-molecule headgroups. However, the residual weights for these four polymer samples at 800 °C were in the range of about 9%, which could be ascribed to the POSS molecule in polymers. As a result, the model functionalization of VPOSS-PCL with small functional groups was conveniently achieved by thiol-ene ligation, leading to single-tailed giant surfactants with polar heads modified with hydroxyl groups, carboxylic acids, or amine groups.

**Figure 9:**
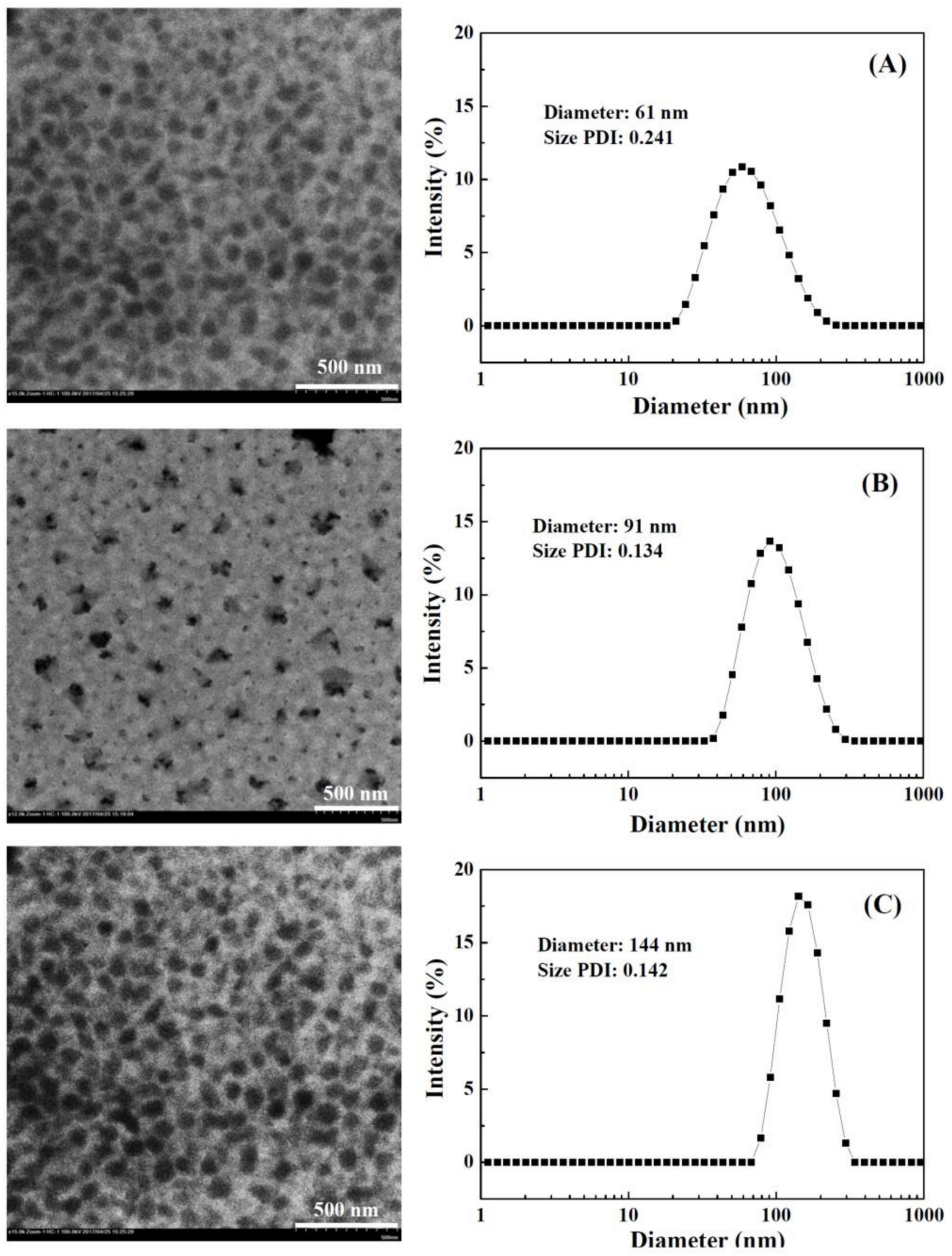
TEM images (scale bars represent 500 nm) and plots of particle size distributions of nanoparticles formed from (**A**) 7OH-POSS-PCL, (**B**) 7COOH-POSS-PCL, and (**C**) 7NH_3_Cl-POSS-PCL in aqueous solution with a concentration of 0.2 mg/mL.

As a size-amplified counterpart of small-molecule surfactant, the single-tailed giant surfactants were composed of hydrophobic polymeric chain and hydrophilic headgroups. Therefore, the giant surfactants should also self-assemble in aqueous solution to form nanoparticles. The preliminary self-assembly study of the present PCL-based giant surfactants was carried out. The morphology, average particle sizes, and size polydispersity indices (size PDIs) of the nanoparticles self-assembled from various polymers were investigated by DLS and TEM measurements, and the results are displayed in Figure 9. One can find that the giant surfactants mainly formed spherical nanoparticles, and the corresponding size distribution curves of the nanoparticles measured by DLS show monomodal peaks.

## 4. Conclusions

In summary, we have provided a facile approach to synthesize giant surfactants containing a poly(ε-caprolactone) (PCL) tail and a POSS head with diverse surface functionality by the combination of ROP reaction and thiol-ene “click” chemistry. The polymers were fully characterized by ^1^H NMR, ^13^C NMR, GPC, MALDI-TOF mass spectrometry, and TGA. Moreover, the preliminary self-assembly investigation demonstrated that these giant surfactants can form nanospheres with different sizes in aqueous solution. This synthetic way demonstrates a click philosophy by constructing straightforward and diverse structures from an easily available precursor, using a simple set of high-efficiency chemical transformations. It is expected that the methodology should be easily extended to other polymer systems and functional headgroups for fine-tuning the interaction parameters of giant surfactants.

## Figures and Tables

**Table 1:** Characterization results of VPOSS-PCL.

Entry	Samples	*M*_n, NMR_^a^	*M*_n, GPC_^b^	*M*_w_/*M*_n_^b^
1	VPOSS-PCL-1	2300	3800	1.09
2	VPOSS-PCL-2	4100	5000	1.07
3	VPOSS-PCL-3	5400	7700	1.11

^a^ Calculated on the basis of ^1^H NMR analysis in CDCl_3_; ^b^ determined by GPC analysis with THF as the eluent.
